# A scoping review of the ethical impacts of international medical electives on local students and patient care

**DOI:** 10.1186/s12910-023-00998-7

**Published:** 2024-01-03

**Authors:** Magdalena Chmura, Shobhana Nagraj

**Affiliations:** 1 Medical Sciences Division, University of Oxford, Oxford, UK; 2 Oxford University Global Surgery Group, University of Oxford, Oxford, UK; 3 Health Systems Collaborative, Centre for Global Health Research, Nuffield Department of Medicine, University of Oxford, Oxford, UK

**Keywords:** Ethics, Electives, Medical students, LMICs

## Abstract

**Background:**

International electives are often considered a valuable learning opportunity for medical students. Yet, as travelling to lower and middle income countries (LMICs) becomes more common, ethical considerations of such practices emerge. We conducted a scoping review to assess the extent to which five ethical themes were addressed in existing literature about electives, with the aim of investigating the ethical impacts of medical student electives on local resources, patients and clinicians in LMICs.

**Methods:**

We systematically searched PubMed, Global Health and Embase databases using the search terms “(ethics) AND (medical electives)”. Thematic content analysis was undertaken using a combination of deductive and inductive themes. The deductive themes included: exceeding clinical competence, use of limited local resources, respect for patients and local culture, collaboration with local community/colleagues, and one-sided benefits in partnership. In addition, we also allowed for emerging themes within the data, and conducted a narrative synthesis of the results.

**Results:**

A total of 37 papers discussed ethical issues relating to medical student international electives to LMICs. More publications were written from the medical student perspective (n = 14), than by the host-institution (n = 5), with nearly half written from third-party perspectives (n = 18). Negative impacts on local host students and impact upon patient care were identified as additional ethical considerations.

**Conclusions:**

Our review demonstrated that while there is a degree of awareness in the existing literature of the potential negative impacts of medical electives to local LMIC students’ access to medical education and patient care, continued work is needed to ensure equitable partnerships. We recommend that these ethical themes should be further explored in pre-departure elective teaching courses and post-elective debriefs to increase medical students’ awareness of the impact of their presence on host communities.

**Supplementary Information:**

The online version contains supplementary material available at 10.1186/s12910-023-00998-7.

## Introduction

Medical electives refer to undergraduate placements undertaken by medical students, often in final year of university. The medical elective is embedded in the curriculum of many universities around the world, and in the UK comprises of a period of 4–12 weeks. The Medical Schools Council (MSC), UK, states that an elective “provides the opportunity to develop new skills, increase awareness of social and cultural issues, and be exposed to different healthcare settings” [1]. Electives can also benefit students by increasing their understanding of tropical medicine, public health and social determinants of health [2]. The independent and flexible nature of electives allows students to organise placements in a range of specialties and locations.

A 2002 survey of medical students from UK universities found that 40% of students travel to low and middle income countries (LMICs) for their elective [3]. However, as the MSC notes, “ensuring that the placement is beneficial to both the student and the host is a complex task” [1]. Over the last few decades, medical student participation in global health experiences has increased; in the US the number of students participating in international electives increased from 6% in 1984 to 30% in 2010 [4]. With increasing popularity of international travel for electives, ethical considerations of these practices emerge [5]. During pre-elective teaching courses and post-elective debriefs, students are encouraged to think and reflect on elective experiences, interactions with the local community, and any emerging ethical dilemmas [5].

Several publications, discussed below, have addressed ethical issues associated with travel, in particular to LMICs, written from the standpoint of students, host institutions, and third parties, however ethical considerations are not always explicitly embedded into the discourse around medical electives and their impacts on host institutions in LMICs. In a 2014 literature review, a social accountability framework was used to assess how pre-departure training addressed ethical issues around electives [6]. One of the findings was that electives are focused primarily on benefiting visiting students from wealthy countries while providing no benefit to the host [6], a finding identified in other literature [7]. There may actually be a negative impact on host institutions, as students often lack the necessary training to carry out some tasks [8] or lack the language skills to contribute to the placement in a helpful way [2, 9]. As a result, visiting students may require more supervision and direct resources and staff away from their prior clinical duties [2, 10, 11].

Lack of supervision at the host institution can also be an ethical and legal issue. Rowthorn and colleagues describe cases of students acting without adequate supervision and outside the scope of their competence and training, including instances of performing a lumbar puncture, delivering a baby, and prescribing medication [12]. Other issues can stem from the students’ culture shock, or a lack of contextual knowledge about local culture and customs. This can impact the students’ ability to appropriately obtain informed consent from patients, appropriately manage issues around confidentiality and patient privacy, or work effectively with colleagues [2, 13].

To ensure ethical and effective partnerships between visiting students and host institutions it is vital to identify ethical dilemmas, address them and implement constructive change [7]. A systematic review of the literature on educational interventions by Rahim and colleagues identified 13 ethically complex situations which students should be prepared to manage during their elective and identified educational interventions to prepare students for these ethical challenges [14]. When designing the methods of this scoping review (as outlined below), five focus themes were formed which encompassed ethically complex situations identified by Rahim and colleagues, in conjunction with other ideas identified in background reading. The data were then grouped into papers written from host-perspective and student-perspective in order to evaluate how the different themes are addressed within different types of literature, and emerging themes of negative impacts on local students and patients were also discussed.

This systematic scoping review aimed to identify the inclusion of ethical considerations within the existing literature about medical student electives, to address the research question: What are the ethical impacts of medical student electives on local resources, patients and clinicians in LMICs?

## Methods

The scoping review was carried out in December 2021 using established methods outlined by Arksey and O’Malley [15] and PRISMA guidance [16]. The methods consist of a five-stage framework: identifying the research question (stage one), identifying the relevant studies (stage two), study selection (stage three), charting the data (stage four), and finally collating, summarising and reporting the results (stage five). Once the research question was identified (stage one, above), we conducted an advanced search of three databases (PubMed, Global Health, and Embase) between 2000 and 2021 using the search terms and the medical subject heading terms “(ethics) AND (medical electives)” (stage two). Inclusion criteria were: papers written in English, discussing international electives to LMICs, undertaken by medical students, and including discussion of ethics. Studies were excluded if they discussed electives in a high-income country (HIC), were undertaken by doctors or trainees rather than medical students, and the papers contained no discussion of ethics (stage three).

Included papers were read by one author (M.C.), and thematic content analysis was conducted using both deductive (based on five pre-determined themes) and allowing for inductive elements (emerging during the review process) (stage four).

The pre-determined themes were adapted from those areas of importance identified by Rahim and colleagues on the educational interventions preparing students for ethical challenges of electives [14], as well as on areas identified during background reading. As there was some overlap between the themes, for the purpose of this study and to facilitate charting data, we focused on 5 ethical themes, shown in Table 1 to form the basis of our thematic content analysis. In addition to these five deductive themes, we maintained an open approach for any emerging ethical themes resulting from the data analysis. We synthesized these data into a narrative synthesis of results (stage five).

**Table 1:** Focus themes and examples of theme components adapted from Rahim et al. [14]

Theme	Examples of theme components
Exceeding clinical competence	Working outside of the scope of one’s competence or training Working without adequate supervision Management of unrealistic patient or staff expectations with regards to skills
Use of limited local resources	Use of disposable equipment Financial implications of hosting students Use of clinician and staff time for supervision, teaching, translating
Respect for patients and local culture	Obtaining informed consent Managing privacy and confidentiality
Collaboration with local community and colleagues	Working effectively with colleagues in LMIC Working with local community which may have different social norms
One-sided benefits in partnership	Lack of opportunities for students from LMICs to travel to country from which they are receiving visiting students on electives Lack of teaching provided to students from LMIC, despite host institution providing teaching to visiting students

## Results

### Literature selection results

**Fig. 1:**
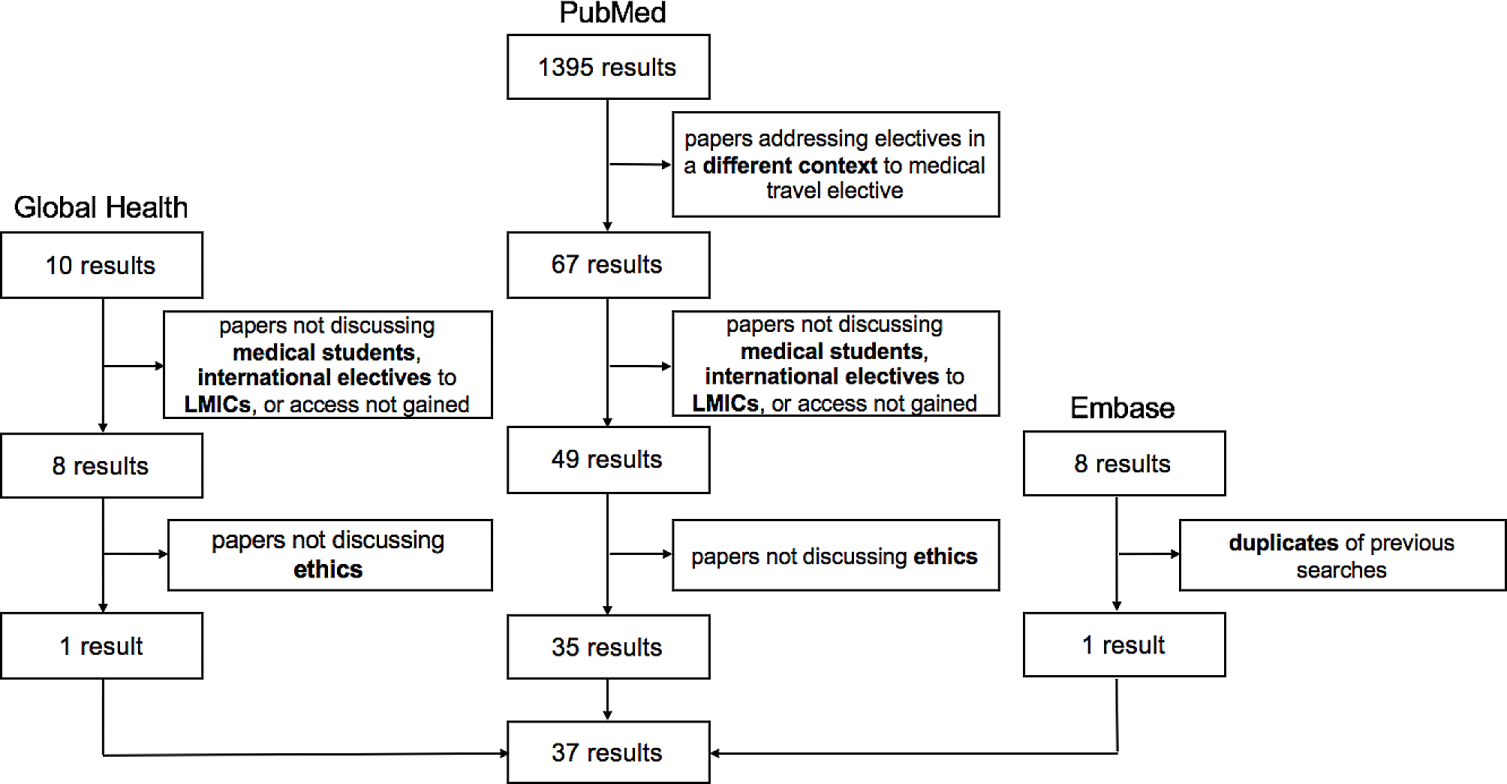
PRISMA flow chart of the review

The results of the advanced search of databases is shown in the PRISMA diagram Fig. 1. The searches yielded 1413 studies. Following removal of duplicates and studies not meeting inclusion criteria, 37 papers were included in the scoping review. Final papers included in the review are listed in supplementary Table S1.

### Overview of the selected studies

Travel destinations were mostly based in Sub-Saharan Africa (including Malawi [17, 18], Zimbabwe [17], Zambia [18], Uganda [2, 19, 20] Tanzania [18, 20], Botswana [21, 22], Ghana [23, 24], Benin [25], Kenya [17, 26] and South Africa [19, 26, 27]), but also included other LMICs such as Solomon Islands [8, 28, 29] and India [2, 17, 22, 26]. Students described in the studies mostly came from the USA (including Michigan [24] and Pennsylvania [21]), the UK (including London [17], Oxford [30], and Birmingham [31]), and Canada [2, 22, 26, 32].

There were a variety of types of papers, including interviews with students and clinicians, descriptions and evaluations of existing teaching and exchange programs, as well as editorials, literature reviews and a letter to the editor. For the purpose of this analysis, rather than being grouped by the type of journal article, the papers were grouped by the “perspective” on which they were based. The first group was publications based on interviews and reflections of students returning from electives (14 papers) [8, 17–20, 22, 24, 26, 27, 29, 31–34]. The second group consisted of publications written by members of host institutions in LMICs, or based on interviews with them (5 papers) [2, 25, 28, 35, 36]. The third group was publications which were produced by neither of these groups, consisting primarily of editorials, systematic reviews, and guidelines written by universities (18 papers) [5–7, 9–14, 21, 23, 30, 37–42].

### Summary of main results

**Fig. 2:**
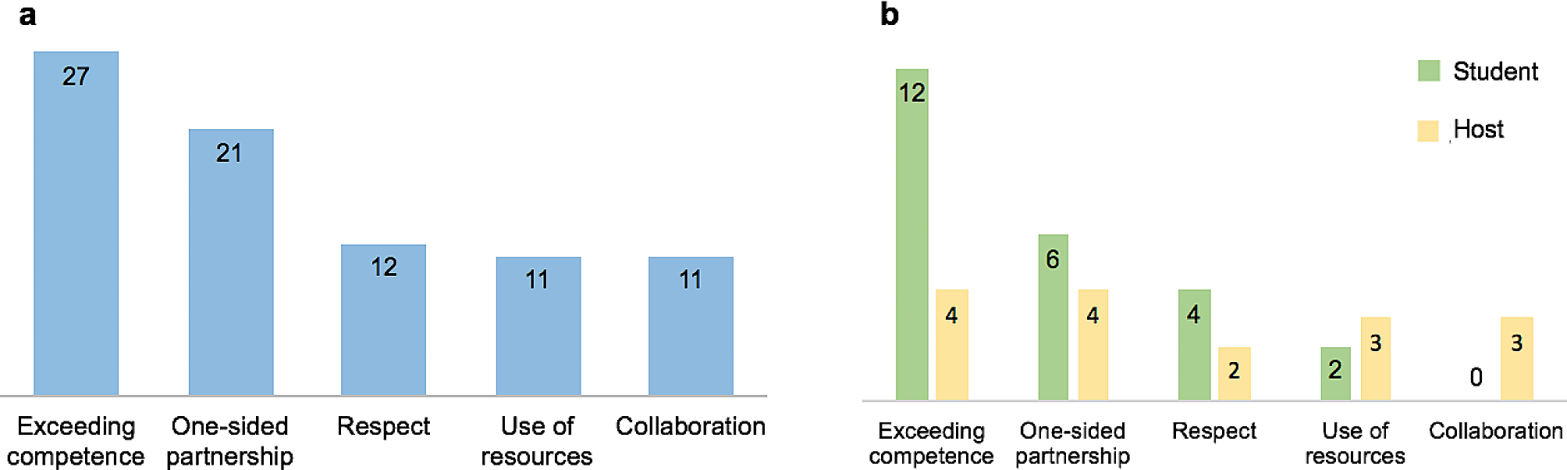
**a** Number of papers addressing each focus theme, **b** Number of papers from student and host perspective addressing each focus theme

Papers were analysed with respect to the five focus themes, grouped visually and the number of papers addressing each of these themes is illustrated in Fig. 2A. Many papers mentioned more than one theme. To see which ethical themes were predominantly explored by travelling medical students and which by host institutions, these data were further broken down and grouped visually to show the number of student and host papers addressing each theme, as shown in Fig. 2B. Of note, there were fewer host-perspective than student-perspective publications.

### Exceeding clinical competence

As seen in Fig. 2A, exceeding clinical competence was the most commonly mentioned of the pre-defined themes. In interviews with UK medical students who travelled to Solomon Islands for a 4-week elective, a key finding was that students reported being asked to carry out tasks which they would not be asked to perform in the UK [8]. The attitudes towards whether or not it is ethical to act outside of one’s scope of competence were also mixed. In a study by Wiskin and colleagues, 379 students returning from electives were interviewed and 49% respondents said it is “more acceptable to work outside of the UK parameters” in LMICs [31]. In some studies, respondents reported that they “chose to go on an [international health elective] because they anticipated having more opportunities to practise clinical skills” [26]. By contrast, in other studies students expressed difficulty assessing whether they were competent to carry out a procedure, especially in an unfamiliar setting, which made them reluctant to carry out tasks [8, 25].

### One-sided benefits in partnership

Some papers discussed the need for establishing bidirectional travel programmes as opposed to unilateral ones [2, 24], and identified administrative, financial and language barriers which can prevent students from LMICs from travelling to a HIC) [38]. The importance of exchange of teaching and ideas between the institutions was also discussed [21, 30, 42]. One report of an elective partnership between UPenn and a hospital in Botswana suggested that this can be done through including the host institution in research, abstracts, and posters [21], while another paper focused on use of peer-to-peer teaching [42]. Some papers found that the lack of benefit to the host institution from accepting visiting students is largely due to inadequate preparation of visiting students prior to embarking on their elective placement, and the short duration of placement [30]. Borah and colleagues evaluated a year-long elective partnership between Loyola University Chicago Stritch School of Medicine and a clinic in Bolivia, and found that the longer length of stay mitigated many ethical challenges associated with travel to LMICs. It allowed the students to “become integral and accountable members of a full-functioning healthcare team” and “long-term responsibilities may decrease the potential burden that they could otherwise impose upon their host site”, which touches on the themes of “one-sided benefits in partnership”, “use of limited local resources”, and “collaboration with local community and colleagues” [11].

### Respect for patients and local culture

A common point discussed within the theme of “respect for patients and local culture” was the idea of consent. In a paper based on interviews with students after electives, a student reported that she wished to obtain consent from a patient before examining her abdomen but did not do so because she felt “constrained within the local medical hierarchy”, while another respondent discussed “the idea that privacy seemed not to exist” [26]. In a series of interviews with members of host institutions, many respondents reported that “students had higher standards than expected” and one respondent explained that students “sought informed consent before practising procedures such as injections or physical examinations” which was not routinely done in their institution [25]. Willott and colleagues explained that this discrepancy between expectations in obtaining consent can stem from practical limitations, as “contact time with patients is sometimes so limited that there is no opportunity for discussion or any meaningful choice other than to forgo treatment, as typically happens in public hospitals in India” [23].

### Use of limited local resources

Although overall in this review there were more student papers than host, more host papers than student papers mentioned this particular theme. One paper detailed a “Responsible Elective” programme set up by Dundee Medical School which aimed to promote an ethical partnership with a centre in Malawi, with the expectation that students will fundraise for the centre. The aim was for students to recognise “that these experiences, particularly in [low-income countries], can consume already limited resources at host hospitals with limited benefits for them” [23]. In another study, clinicians from host institutions said that financial support from partner institutions could help offset some of the costs of hosting elective students, such as hiring translators [35]. In addition to financial resources, some papers recognised the use of other resources. Several papers identified that elective students use the supervising clinician’s time [2, 11, 26], and that presence of elective students increases clinician workload [25].

### Collaboration with local community and colleagues

Analysis of “collaboration with local community and colleagues” revealed the importance of elective students being educated about ethics and following local laws. In an overview of the ethical and legal frameworks of short term experiences in global health, Rowthorn and colleagues stressed the importance of working together with local community leaders and that “university leaders and advisors must educate their students about ethical engagement with communities” [12]. In another paper, the role of students’ pre-departure briefings and guided reflections was raised as a way to ensure ethical behaviour is promoted and students are seeking genuine collaboration with the local community [5]. It also addressed the notion that policies around professional standards during electives should be developed and “these policies should be created in collaboration with host institutions” [5]. In a series of interviews with members of host institutions, the importance of long-term collaboration was highlighted, whereby elective students could establish networks within the local community, and return after graduating as researchers or teachers [36].

### Inductive themes emerging from the papers: negative impacts on local students and patients

The first theme which emerged during our review was “negative impact on local students”. In a multicentre study by Kumwenda and colleagues, based in seven elective sites in sub-Saharan Africa, there were reports of difficulty making schedules for elective students which would not interfere with ongoing teaching, and problems emerging “when clinical staff assigned to supervise visiting students were also expected to teach local students” [35]. Another paper by Willott and colleagues reported that “clinicians and others in LMIC settings can devote disproportionate amounts of their time teaching and concerning themselves with the visiting HIC medical students on elective to the possible detriment to their own students” [23]. This idea was also discussed by Elit and colleagues, who in a series of interviews with elective students found that “health care staff often focused on the needs of the Western medical students” [26]. Interestingly, the majority of papers written from host perspective mentioned the negative impact of electives on local students [2, 35, 36]. By contrast, in papers written from visiting student perspective, this theme was only addressed in one [26].

The second theme which emerged was “negative impact on patient care”. When looking at the papers addressing limited local resources, six of them mentioned that visiting students may have a detrimental effect on patient care [2, 9–11, 25, 26]. In a series of interviews with supervisors of elective students in 22 countries, Bozinoff and colleagues found that “host supervisors identified potential harms to patient care that might arise in the presence of visiting medical students, including harms associated with differing standards of care, longer wait times, lack of cultural competency, and language barriers between students, patients and staff” [2]. In addition to this, papers found that elective students can negatively impact care because “adequately supervising them diverts human resources from providing care” [10]. Interestingly, two host-perspective papers mentioned negative impact on patient care [2, 25], while only one student-perspective paper did [26].

## Discussion

Our scoping review demonstrated that there is some degree of awareness in the literature of the ethical shortcomings of medical electives. These results support those found by Rahim and colleagues [14], illustrating that with increased international travel to LMICs for electives, there has been ongoing discussion in published work about ethical implications of such practices. In addition, this data reveals that while the largest proportion of papers published in English in the three databases and meeting the inclusion criteria is by third parties, the second largest group of publications is written based on interviews and discussions with students returning from electives. There is a sense that host perspective is missing from the narrative, as literature written by members of host institutions makes up a minority of these papers (only 5 of the 37 papers included in this review). This is in line with findings from a previous literature review by Wallace and colleagues, who found that “research has focused primarily on the student experience, rather than also eliciting the perspectives of local physicians, and community organizations and members” [6]. This highlights a gap in the current conversations around ethics of electives, and should prompt future research to include the point of view of members of host institutions.

The first part of the review looked at the main ethical themes addressed within literature. It appears that there may be a mismatch between what the host institutions regard as the key themes and what the visiting students identify and reflect upon. Within student-perspective literature “exceeding clinical competence” is addressed twice as much as the second-most addressed theme “one-sided benefits in partnership”. In a study based on interviews with Canadian medical students after their electives, authors reported “moving beyond one’s scope of practice” as one of 5 main ethical themes [26]. The discussion of other themes included students’ acknowledgement of the placement’s benefit to their learning, yet there was limited discussion of how to make the placement beneficial for host institutions.

By contrast, in host-perspective literature the two themes are addressed in the same number of papers, which could imply that both themes are widely recognised by the hosts (however, the small number of host-perspective papers may limit the extent to which one can conclude this). Renaud-Roy and colleagues conducted interviews with Beninese health care professionals who hosted Canadian students for electives, and found that the supervisors expressed little concern about students’ willingness to act outside of their competence [25]. However, many respondents expressed that lack of reciprocity and the unilateral nature of exchange are shortfalls of the partnership [25]. One physician interviewed as part of a study by De Visser and colleagues in Uganda and Tanzania concluded that “the relationship has to be reciprocal…it’s not ethical if it’s not reciprocal” [36].

The mismatch between the types of papers addressing these two themes could suggest that students do not adequately recognise the ethical themes of one-sided benefit, to the extent that host institutions do. It may be that by focusing post-elective reflections on the ethical theme of exceeding competence, students are missing other important themes which are acknowledged within the host community.

One of the inductive themes which emerged was that there may be a negative impact of electives on local medical students and their education. This theme is also mentioned more commonly in host-perspective literature than in student-perspective literature. In a study looking at interviews with clinical staff from Malawi, Zambia, and Tanzania, it was found that almost all hospitals which receive elective students also have their own medical or nursing schools [35]. Some clinicians reported encountering problems when supervision of visiting students interfered with ongoing teaching, and other studies expressed that this was to the possible detriment of local students [23]. The second inductive theme which emerged was that there may be a negative impact of elective students on patient care. One student-perspective study presented a contrary idea, suggesting that students can take pressure off busy doctors by taking care of straightforward patients and improving triage [8]. However, other studies including host-perspective studies found that visiting students can take skilled staff away from their clinical duties [2, 9–11, 26].

These findings collectively imply that visiting students are not recognising the detrimental impact they may have on other students in LMICs, and on the limited resources, and as a consequence are not addressing the local impact of these issues on students, clinicians and patients.

It is important to note a limitation of the study and its findings: the databases and papers included were all in English, and did not include publications in other languages. This study design could potentially exclude many studies and publications in other languages, which could disproportionately affect papers written by members of host institutions in LMICs. The low number of host-perspective literature identified by the method is a limitation which needs to be considered when drawing conclusions. Another limitation is that there was only one author (M.C.) reading the papers and thematic content analysis was conducted by a single reviewer which might have introduced bias in the themes. We reduced this bias by including predefined themes and through discussion with a wider team of colleagues.

Though pre-departure teaching and post-elective reflections can be a valuable way for students to become more aware of ethical shortcomings of their elective, it is apparent that not all themes are being adequately addressed yet. Perhaps the goal should not simply be for students to reflect on what is important from their perspective, but instead to acknowledge what the host institutions regard as the most pertinent issues. Considering the common themes identified in host literature, three changes have been suggested which could improve the equity of elective partnerships, implement bidirectional exchange and increase benefit to host institutions.

Firstly, visiting students could aim to increase the positive impact on the host institution during their stay, to limit or offset the negative impacts on resources, local students, and patients. Shah and colleagues looked at forming a framework for short term experiences in global health, and suggested that the participants should shift their focus from “fixing or saving the world” to ensuring the impact on host community is prioritised [43]. Borah and colleagues evaluated a year-long elective programme and its advantages over the more traditional short-term electives. Authors suggested that longer duration of placements benefits the host site and patients, as it strengthens the relationship between the two institutions, and allows establishment of common goals [11]. Based on evaluation of other programmes, it was suggested that increasing the training and support for visiting students from their institution can increase their ability to benefit the host hospital [37].

Secondly, steps could be undertaken by institutions to make exchange programmes mutually beneficial. This could be achieved through including members of host institutions in research collaborations, abstract or poster presentations [21], or peer-to-peer teaching [42]. Moreover, interviews with members of host institutions showed that the opportunity to send their own students to the institutions from which they are receiving visiting students would be the main improvement [36]. For instance, in the USA, more than a fifth of the medical schools with exchange programmes do not accept their collaborators’ students onto their own campus [38]. Some institutions have reciprocal arrangements in place, with scholarships to fund travel costs, and perhaps universities could strive towards this model [23]. Finally, the ethical themes addressed in this study should be further explored in pre-departure teaching courses, and post-elective discussions to allow exchange of ideas around ethics from host and student perspective. The use of online resources and book-length manuals covering ethics and elective preparation has also been suggested [44]. Perhaps designing such resources together with members of host institutions could be a useful tool in supplementing ethics learning and ensuring the host perspective is further incorporated into pre-departure teaching.

## Conclusion

This study aimed to answer the question “what are the ethical impacts of medical student electives on local resources, patients and clinicians in LMICs?”. While there is some discussion in the literature of the ethical implications of electives, there is a mismatch between what students and hosts emphasise in their writing. The possible negative impact of their presence on local students’ education and on patient access to care are examples of ethical themes which are not adequately addressed by students on their electives. Future efforts should focus on finding ways of implementing mutually beneficial changes into elective programmes, as well as incorporating these ethical themes into the pre-departure training and post-elective debriefs and publications, to increase students’ awareness of the impact of their presence on the host community.

### Electronic supplementary material

Below is the link to the electronic supplementary material.

Supplementary Material 1

## Data Availability

All data generated or analysed during this study are included in this published article and its supplementary information files.

## Abbreviations

HIC: High Income Country
LMIC: Lower and Middle Income Country
MSC: Medical Schools Council
