# Supplementary material for: A scoping review of the ethical impacts of international medical electives on local students and patient care
Source: BMC Med Ethics. 2024 Jan 3;25:5. doi: 10.1186/s12910-023-00998-7 (PMC10765728; doi:10.1186/s12910-023-00998-7)
Supplement: Supplementary file 1 — Supplementary Material 1 [file 12910_2023_998_MOESM1_ESM.docx]

***Table S1:*** *Papers included in the scoping review*

| Title | Year | Authors | Study details |
| --- | --- | --- | --- |
| [Practising on the poor? Healthcare workers' beliefs about the role of medical students during their elective.](https://pubmed.ncbi.nlm.nih.gov/15681677/) | 2005 | S J J Radstone | Questionnaire administered to healthcare staff in one hospital in Solomon Islands. Identified discrepancies in what the host country staff think medical students are qualified to do and what they are allowed to do, leading to students exceeding their competence in LMICs.  Written from host standpoint.  Countries of interest: Solomon Islands |
| [Electives: isn't it time for a change?](https://pubmed.ncbi.nlm.nih.gov/19161481/) | 2009 | Jon Dowell, Neil Merrylees | Guide analysing roles of an elective, how it contributes to training and how it can be improved to maximise learning opportunities. Addressed support during placement and ethical challenges including barriers for students from LMICs traveling to HICs. |
| [Medical electives in South Africa.](https://pubmed.ncbi.nlm.nih.gov/20218475/) | 2009 | [Matthew Anthony Kirkman](https://pubmed.ncbi.nlm.nih.gov/?term=Kirkman+MA&cauthor_id=20218475) | Reflection written by a medical student after an elective. Discussed the ethical issues around exceeding competence while on electives.  Written from student standpoint.  Countries of interest: South Africa, UK |
| [Worrying behaviour on electives. Practising on the poor?](https://pubmed.ncbi.nlm.nih.gov/19208707/) | 2009 | [Samantha J J Radstone](https://pubmed.ncbi.nlm.nih.gov/?term=Radstone+SJ&cauthor_id=19208707) | Letter to editor about personal experience of seeing medical students acting outside competence. Argued that host institutions should be made aware of what the visiting students are allowed to do in HICs in order to ensure they do not exceed their competence.  Written from student standpoint.  Countries of interest: Solomon Islands |
| [International health electives: thematic results of student and professional interviews.](https://pubmed.ncbi.nlm.nih.gov/20636587/) | 2010 | Andrew Petrosoniak, Anne McCarthy, Lara Varpio | Analysis of 20 interviews with healthcare professionals and trainees who went on electives. Discussed the idea of medical tourism and found that participants’ awareness of this issue was low.  Written from student standpoint.  Countries of interest: Canada |
| [Ethical issues encountered by medical students during international health electives.](https://pubmed.ncbi.nlm.nih.gov/21649703/) | 2011 | Laurie Elit, Matthew Hunt, Lynda Redwood-Campbell, Jennifer Ranford, Naomi Adelson, Lisa Schwartz | Interviews with 12 students after elective. Identified five themes of ethical issues: uncertainty about how best to help; perceptions of Western medical students as different; moving beyond one’s scope of practice; navigating different cultures of medicine, and unilateral capacity building.  Written from student standpoint.  Countries of interest: Nepal, India, Thailand, Uganda, Ghana, Kenya, Tanzania, South Africa, Honduras, Nicaragua, El Salvador and Venezuela, Canada |
| [Socially responsible approaches to international electives and global health outreach.](https://pubmed.ncbi.nlm.nih.gov/21486355/) | 2011 | Shafik Dharamsi, Jo-Ann Osei-Twum, Matt Whiteman | Report of interactive workshops in preparation for global health projects. Case-based discussions and consideration of sustainability, cultural competency, human dignity, power and privilege, colonialism and imperialism. Concluded that further preparation and global health teaching should be embedded in medical school curricula.  Countries of interest: Canada |
| [The contribution and challenges of electives in the development of social accountability in medical students.](https://pubmed.ncbi.nlm.nih.gov/21774651/) | 2011 | Deborah Murdoch-Eaton, Andrew Green | Article about impact of electives on students undertaking them. Discussed importance of anticipating intentional and unintentional learning outcomes to ensure benefit to both student and host institution.  Countries of interest: UK |
| [Ethical considerations in International Health Electives.](https://pubmed.ncbi.nlm.nih.gov/21918944/) | 2011 | Rakhi Sinha | Letter to editor in response to the article the article “Effects of International Health Electives on Medical Student Learning and Career Choice.” Addressed the idea that students often work beyond their competence and increase the work burden on host. |
| [Adopting an ethical approach to global health training: the evolution of the Botswana - University of Pennsylvania partnership.](https://pubmed.ncbi.nlm.nih.gov/24072119/) | 2013 | Matthew Dacso, Amit Chandra, Harvey Friedman | Overview of a partnership between a centre in Botswana and UPenn. Discussion of ways to achieve mutually beneficial partnership, such as through including members of LMIC institutions in research, abstracts and posters.  Countries of interest: Botswana, USA |
| [Should trainee doctors use the developing world to gain clinical experience? The annual Varsity Medical Debate - London, Friday 20th January, 2012.](https://pubmed.ncbi.nlm.nih.gov/23433035/) | 2013 | Barnabas J Gilbert, Calum Miller, Fenella Corrick, Robert A Watson | Article about the Varsity medical debate, discussion of effect of electives on participant and host institution. Concluded that organisation and planning are key to ensuring patient safety and a positive impact.  Countries of interest: UK |
| [The ethics and safety of medical student global health electives.](https://pubmed.ncbi.nlm.nih.gov/25341214/) | 2014 | Evelyn M Dell, Lara Varpio, Andrew Petrosoniak, Amy Gajaria, Anne E McMcarthy | Interviews of 23 participants who went on electives, with pre departure training and post experience briefing. Addressed resource disparities and provision of care; navigating clinical ethical dilemmas; and threats to trainee safety.  Written from student standpoint.  Countries of interest: Botswana, India, Canada. |
| [Pre-departure training and the social accountability of International Medical Electives.](https://pubmed.ncbi.nlm.nih.gov/25420975/) | 2014 | Lauren J Wallace, Allison Webb | Literature review with a focus on using the social sustainability framework, analysis of the effectiveness of pre-departure training. Found that there is limited discussion in literature of pre-departure training and argued that global health education should be integrated into medical school curricula.  Countries of interest: UK |
| [Western medical students' experiences on clinical electives in sub-Saharan Africa.](https://pubmed.ncbi.nlm.nih.gov/24807435/) | 2014 | Ben Kumwenda, David Royan, Paige Ringsell, Jon Dowell | Interviews with 29 students who travelled to 7 hospitals in Sub Saharan Africa. Discussion of choice of location, motivations for travel, and levels of supervision. Found that there was adequate supervision of students and inappropriate practice did not occur.  Written from student standpoint.  Countries of interest: Malawi, Tanzania, Zambia, USA, UK, Netherlands, Australia, New Zealand, Denmark |
| Toward reciprocity: host supervisor perspectives on international  medical electives. | 2014 | Nikki Bozinoff, Katie P Dorman, Denali Kerr, Erica Roebbelen, Erin Rogers, Andrea Hunter, Tim O'Shea, Christian Kraeker | Study based on a questionnaire of 39 host country supervisors in 22 countries, addressed health risks to students, costs assumed by students, potential for students to experience culture shock Identified reciprocity and bidirectional exchange as a way to improve partnerships.  Written from host standpoint.  Countries of interest: Angola, China, Ethiopia, Ghana, Guyana, India, Kenya, Nepal, Rwanda, South Africa, Uganda, Vietnam, Australia, Barbados, Ireland, Israel, Kuwait, Saudi Arabia, Trinidad/Tobago, UK, USA, Canada |
| Electives in undergraduate medical  education: AMEE Guide No. 88. | 2014 | Andrew Lumb, Deborah Murdoch-Eaton | Case studies of students who undertook electives. Concluded that pre-departure preparation is an essential component of ensuring electives meet learning objectives.  Written from student standpoint. |
| [Medical electives in sub-Saharan Africa: a host perspective.](https://pubmed.ncbi.nlm.nih.gov/25989410/) | 2015 | Ben Kumwenda, Jon Dowell, Katy Daniels, Neil Merrylees | Multicentre study involving interviews at 7 elective sites in sub-Saharan Africa. Generated 4 themes: organisation, pros and cons of hosting, elective environment and clinical supervision, improving elective systems  Written from host standpoint.  Countries of interest: Malawi, Zambia, Tanzania. |
| Preparing students for the ethical challenges on international health electives: A systematic review of the literature on educational interventions. | 2016 | Anika Rahim, Felicity Knights Née Jones, Molly Fyfe, Janagan Alagarajah, Paula Baraitser | Literature review to map range of ethical issues addressed by literature, analyse learning interventions and evaluate evidence of the effectiveness of different approaches. Identified four major themes with thirteen sub-themes. |
| [An ethical partnership model: clinical electives in Africa.](https://pubmed.ncbi.nlm.nih.gov/27072448/) | 2016 | Alice McGarvey, Eric O'Flynn, Sean Tierney | An outline of a new COSECSA partnership with RCSI. Concluded that a successful partnership has been established and that more partnerships like this should be further developed in order to aid future training of medical professionals.  Countries of interest: Ireland |
| [Bidirectional Exchanges of Medical Students Between Institutional Partners in Global Health Clinical Education Programs: Putting Ethical Principles into Practice.](https://pubmed.ncbi.nlm.nih.gov/28283116/) | 2016 | Robert Rohrbaugh, Anne Kellett, Michael J Peluso | Summary of US institutions with structured elective programs to identify whether they have bidirectional programs. Found that many HIC institutions do not have a bilateral exchange programme and discussed barriers preventing students from LMICs from visiting HICs.  Counties of interest: USA |
| [Surgical volunteerism or voluntourism - Are we doing more harm than good?](https://pubmed.ncbi.nlm.nih.gov/28433757/) | 2017 | Faheem Ahmed, Madeline Grade, Carl Malm, Sophia Michelen, Na'eem Ahmed | Editorial about benefits and challenges of volunteering in LMICs to hosts and visitors. Discussed the importance of students being well prepared for electives to ensure they avoid doing more harm than good. |
| Ethical reflection for medical electives | 2018 | Rose Tiller, June Jones | Focus group of Birmingham students was studied during a 4 month placement in Solomon Islands. Identified key issues such as being asked to complete tasks on a higher skill level than in the UK.  Written from student standpoint.  Countries of interest: Solomon Islands, UK |
| Ethical learning on international medical electives: a case-based analysis of medical student learning experiences | 2018 | Gemma Bowsher, Laura Parry-Billings, Anna Georgeson, Paula Baraitser | Case based discussion with 19 participants to identify impact of structured online ethics teaching to support learning during electives. Discussion of uncertainty about norms, perception of western students, scope of practice, navigating different cultures, one sided benefits.  Written from student standpoint.  Countries of interest: UK |
| [Ethical dilemmas during international clinical rotations in global health settings: Findings from a training and debriefing program.](https://pubmed.ncbi.nlm.nih.gov/29094625/) | 2018 | Michael J Peluso, Stacey Kallem, Mei Elansary, Tracy L Rabin | Surveys of 82 medical students before and after electives. Assessed effectiveness of global health ethics training in preparing for ethical challenges. Discussed the ways in which students experience ethical challenges and access support resources.  Written from student standpoint.  Countries of interest: China, South Africa, Uganda, Argentina, Colombia, Peru, Indonesia, Thailand, Chile, Jamaica, Dominican Republic, Mexico, USA |
| [Beyond 'health and safety' - the challenges facing students asked to work outside of their comfort, qualification level or expertise on medical elective placement.](https://pubmed.ncbi.nlm.nih.gov/30029673/) | 2018 | Connie Wiskin, Jonathan Dowell, Catherine Hale | Survey of 379 students returning from electives, discussion of working beyond one's competence. Found that many students were asked to do something outside of their competence.  Written from student standpoint.  Countries of interest: UK |
| [Longitudinal Service Learning in Medical Education: An Ethical Analysis of the Five-Year Alternative Curriculum at Stritch School of Medicine.](https://pubmed.ncbi.nlm.nih.gov/30079441/) | 2018 | Brian F Borah | Evaluation of a year-long elective programme and its advantages over short term electives. Discussed the merits of the model in encouraging development of advocacy, professionalism and social justice.  Countries of interest: USA, Bolivia |
| [Ethical globalization? Decolonizing theoretical perspectives for internationalization in Canadian medical education.](https://pubmed.ncbi.nlm.nih.gov/30018682/) | 2018 | Taqdir Bhandal | Literature review with a focus on ethics of globalisation, colonialism and racism. Identified a lack of discussion in literature about colonial, neoliberal and unjust relations between sites.  Countries of interest: Canada |
| Structured medical electives: a concept whose time has come? | 2019 | Chris Willott, Eva Khair, Roger Worthington, Katy Daniels & A. Mark Clarfield | Conclusions from a discussion about a selection of elective programmes, addressed clinical, professional and social themes. Discussed the benefits of inviting students from LMICs to participate in conferences, and the negative impacts of hosting HIC students on clinician time.  Countries of interest: Ghana, Ethiopia, Israel. |
| [How the Social Contract Can Frame International Electives.](https://pubmed.ncbi.nlm.nih.gov/31550221/) | 2019 | Shailendra Prasad, Fatima Alwan, Jessica Evert, Tricia Todd, Fred Lenhoff | Article exploring short term experiences in global health, addressing competence, accountability, promoting public good. Calls for organisations to address the ethical challenges associated with international electives. |
| [Not Above the Law: A Legal and Ethical Analysis of Short-Term Experiences in Global Health.](https://pubmed.ncbi.nlm.nih.gov/31225956/) | 2019 | Virginia Rowthorn, Lawrence Loh, Jessica Evert, Eleanor Chung, Judith Lasker | Overview of legal framework of short term experiences in global health. Concluded that many of the activities undertaken on short term experiences in global health would be illegal if carried out in the USA.  Countries of interest: USA |
| Healthy, safe and effective international medical student electives: a systematic review and recommendations for program coordinators | 2019 | D. Ashley Watson, Nicholas Cooling & Ian J. Woolley | Qualitative systematic review to identify key themes in publications about electives. The themes identified were: Responsibilities; General policies; Travel advisories; Occupational risk assessment; Funding & finances, Pre-departure training programs; and Post-return debriefing and screening. |
| [Host perspective on academic supervision, health care provision and institutional partnership during short-term electives in global health.](https://pubmed.ncbi.nlm.nih.gov/31875656/) | 2020 | Etienne Renaud-Roy, Nicolas Bernier, Pierre Fournier | Analysis of 30 interviews from Beninese health care professionals who hosted Canadian medical students. Looked at pedagogic relationship, healthcare relationship, institutional relationship. Discussed the lack of opportunities for students from LMICs to visit HICs.  Written from host standpoint.  Countries of interest: Benin, Canada |
| Embedding international medical  student electives within a 30-year partnership: the Ghana-Michigan collaboration. | 2020 | Emma R Lawrence, Cheryl Moyer, Carrie Ashton, Bolade A R Ibine, Nauzley C Abedini, Yaera Spraggins, Joseph C Kolars, Timothy R B Johnson | Retrospective qualitative analysis of student reflections about electives and generation of key themes. Found that long standing bidirectional exchange could allow building of cross-cultural relationships.  Written from student standpoint.  Countries of interest: Ghana, USA |
| Response to: Global health electives:  Ethical engagement in building global health capacity. | 2020 | Alistair J M Reed | Letter to editor about personal experience of seeing medical students acting outside competence without supervision or adequate training.  Written from student standpoint.  Countries of interest: South Africa, Zambia, India, UK |
| [Global health electives: Ethical engagement in building global health capacity.](https://pubmed.ncbi.nlm.nih.gov/32083958/) | 2020 | Adriena De Visser, Jennifer Hatfield, Rachel Ellaway, Denise Buchner, Jeremiah Seni, Wilfred Arubaku, Josephine Nambi Najjuma, Gwendolyn Hollaar | Analysis of 33 interviews and 28 discussions with staff from centres hosting medical students. Found that hosts identified more benefits to visiting students than to host site, as well as challenges and burdens to host.  Written from host standpoint.  Countries of interest: Tanzania, Uganda, Canada |
| [The global variation of medical student engagement in teaching: Implications for medical electives.](https://pubmed.ncbi.nlm.nih.gov/32092102/) | 2020 | Rhys D Wenlock, Michael F Bath, Tom Bashford, Katharina Kohler, Peter J Hutchinson | Analysis of peer-to peer teaching in different countries, discussion of possible role for teaching during electives. Concluded that many students are involved in teaching but that more support and formal training should be provided to them.  Countries of interest: UK, Egypt, Czech Republic, Iraq |
| [Medical electives in sub-Saharan Africa: a 15-year student/NGO-driven initiative.](https://ezproxy-prd.bodleian.ox.ac.uk:5239/ovid-b/ovidweb.cgi?&S=AEFLFPIODNACHKGMKPNJGHNMODLFAA00&Complete+Reference=S.sh.48%7c1%7c1&Counter5=SS_view_found_complete%7c20220144888%7ccagf%7ccaghdb%7ccagh&Counter5Data=20220144888%7ccagf%7ccaghdb%7ccagh) | 2021 | Gianluca Quaglio, Donald Maziku, Marta Bortolozzo, Nicoletta Parise, Chiara Di Benedetto, Alice Lupato, Chiara Cavagna, Ademe Tsegaye & Giovanni Putoto | Interviews with 141 students returning from electives, discussion about the benefits of electives to students. Found that a well-structured, mentored experience can have a positive impact on students’ attitudes and experience.  Written from student standpoint.  Countries of interest: Italy, Angola, Central African Republic, Ethiopia, Mozambique, South Sudan, Uganda, Sierra Leone, and Tanzania |
